# Synthesis, Structural Characterization, and Preclinical Efficacy of a Novel Paclitaxel-Loaded Alginate Nanoparticle for Breast Cancer Treatment

**DOI:** 10.1155/2016/7549372

**Published:** 2016-08-30

**Authors:** Ahmed A. Markeb, Nagwa A. El-Maali, Douaa M. Sayed, Amany Osama, Mohamed A. Y. Abdel-Malek, Amen H. Zaki, Mostafa E. A. Elwanis, James J. Driscoll

**Affiliations:** ^1^Department of Chemistry, Faculty of Science, Assiut University, Assiut 71516, Egypt; ^2^Department of Oncological Clinical Pathology, South Egypt Cancer Institute, Assiut University, Assiut 71516, Egypt; ^3^Department of Medical Biochemistry, Faculty of Medicine, Assiut University, Assiut 71515, Egypt; ^4^Department of Clinical Pathology, Faculty of Medicine, Assiut University, Assiut 71515, Egypt; ^5^The Vontz Center for Molecular Studies, University of Cincinnati College of Medicine, Cincinnati, OH, USA; ^6^Department of Medical Oncology, South Egypt Cancer Institute, Assiut University, Assiut, Egypt; ^7^Department of Radiotherapy, South Egypt Cancer Institute, Assiut University, Assiut, Egypt; ^8^University of Cincinnati Cancer Institute, Cincinnati, OH, USA

## Abstract

*Purpose*. The antitumor activity of a novel alginate (ALG) polymer-based particle that contained paclitaxel (PTX) was evaluated using human primary breast cancer cells.* Materials and Methods*. PTX was combined with ALG in a nanoparticle as a drug delivery system designed to improve breast cancer tumor cell killing. PTX-ALG nanoparticles were first synthesized by nanoemulsification polymer cross-linking methods that improved the aqueous solubility. Structural and biophysical properties of the PTX-ALG nanoparticles were then determined by transmission electron microscopy (TEM) and high performance liquid chromatography (HPLC) fluorescence. The effect on cell cycle progression and apoptosis was determined using flow cytometry.* Results*. PTX-ALG nanoparticles were prepared and characterized by ultraviolet (UV)/visible (VIS), HPLC fluorescence, and TEM. PTX-ALG nanoparticles demonstrated increased hydrophobicity and solubility over PTX alone. Synthetically engineered PTX-ALG nanoparticles promoted cell-cycle arrest, reduced viability, and induced apoptosis in human primary patient breast cancer cells superior to those of PTX alone.* Conclusion*. Taken together, our results demonstrate that PTX-ALG nanoparticles represent an innovative, nanoscale delivery system for the administration of anticancer agents that may avoid the adverse toxicities with enhanced antitumor effects to improve the treatment of breast cancer patients.

## 1. Introduction

Breast cancer is the second most common cancer worldwide, the fifth most common cause of cancer death, and the leading cause of cancer death in women [1]. The global burden of breast cancer exceeds all other cancers and the incidence rates of breast cancer are increasing [2]. Despite significant advances that have improved breast cancer detection and treatment, women continue to die of the disease even when disease is detected at an early stage. While locoregional treatment has improved, there has been little progress to improve the diagnosis and treatment of patients with significantly advanced disease. At present, there are limited curative options for patients with disease that has advanced beyond regional nodes. Novel treatment strategies, such as nanoparticle drug delivery systems, offer the opportunity to enhance the delivery of anticancer therapeutics into breast cancer cells and may eventually improve patient outcomes.

Nanoscale drug delivery systems formulated from biocompatible and biodegradable polymers constitute an evolving approach to improve drug delivery and tumor targeting [3]. ALG particles offer promise as drug delivery nanoparticles because of the biocompatibility, water solubility, and bioadhesive properties that improve drug-binding.

Advantages of ALG also include specific uptake by tumor cells, nontoxic effect on healthy tissue, and accumulation in tumor tissue with demonstrable antitumor efficacy. PTX is a microtubule-stabilizing agent that promotes tubulin polymerization causing cell death by disrupting the dynamics needed for proper cell division. PTX is poorly soluble in aqueous solutions but soluble in organic solvents, for example, alcohols, which lends itself to advanced formulation strategies. The currently available formulation of PTX includes Cremophor EL (polyethoxylated castor oil) and ethanol for solubilization. However, Cremophor EL generates unwanted toxic effects as well as unwanted side effects, for example, hypersensitivity reactions, nephrotoxicity, and neurotoxicity [4]. Biodegradable nanoparticle formulations using poly(lactic-co-glycolic acid) (PLGA) have shown comparable activity to traditional formulations with much faster rates of administration [5]. PTX could be incorporated at nearly 100% efficiency using nanoprecipitation with acetone and PLGA. These same nanoparticles ranged in diameter from 117 to 160 nm and* in vitro* released over 50% of loaded drug over the first 24 h with a much slower release rate of release over the next 96 h. Cellular studies showed up to a 70% loss of viability in NCI-H69 human small cell lung cancer cells at levels as low as 0.025 *µ*g/mL. Another group has added vitamin E TPGS (D-*α*-tocopheryl polyethylene glycol 1000 succinate) as an emulsifier and matrix component to PLGA nanoparticle for PTX release. PSA-PEG nanoparticle containing PTX as a model drug has also been prepared and evaluated by nanoprecipitation methods [5–8].

Cancer nanotechnology is emerging as a new field of interdisciplinary research expected to advance cancer detection, diagnosis, and treatment [9]. NK105 is a PTX-containing polymeric micelle that consists of poly(ethylene glycol) chemically linked to poly(aspartate) [10–12]. NK105 is well tolerated and demonstrates antitumor efficacy against gastric cancers. Genexol-PM® (Samyang Pharmaceuticals, Daejeon City, Korea) is another polymer-based micelle that contains PTX [12]. In a phase I study, Genexol-PM was used to deliver PTX to tumors without additional adverse toxicity and had an increased response rate in metastatic breast cancer patients compared to treatment with Taxol alone [13–15]. Poly(L-glutamic acid)-PTX conjugate (CT-2103) and poly(L-*γ*-glutamyl-glutamine)-PTX nanoconjugate (PGG-PTX) are also PTX-containing polymers being investigated for anticancer activity [16–22]. PGG-PTX has shown antitumor activity* in vivo* that was superior to Abraxane® (Abraxis Bioscience, Summit, NJ, USA) in some mouse models. The pharmacokinetic and tissue distribution of PGG-PTX was improved over PTX alone and the level of active free PTX in the plasma and tumor was increased compared to Cremophor-ethanol mixtures of PTX in mice bearing lung cancer xenografts [17].

**Figure 1:**
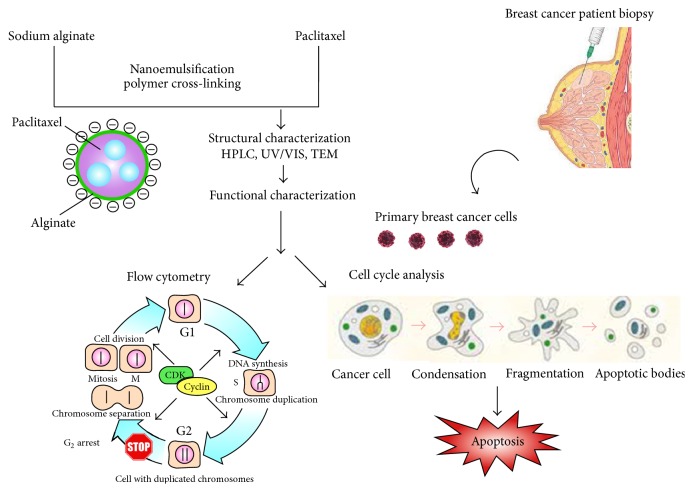
This figure illustrates the design, synthesis, isolation, and functional characterization of paclitaxel-loaded alginate nanoparticles.

We hypothesized that a PTX-micelle conjugate could create a novel class of antitumor nanoparticles with improved therapeutic index compared to that of PTX alone. Here, we generated ALG-PTX nanoparticles and demonstrate their ability to mediate efficient drug delivery and killing of breast cancer cells. To achieve this goal, ALG-PTX was synthesized and prepared in nanoparticles using a simple emulsion method (Figure 1). The resulting nanoparticles were then compared using UV/VIS methods, HPLC-fluorescence detection, and TEM with unmodified PTX. Our results clearly demonstrate the potential of ALG-PTX nanoparticles to efficiently target and eradicate breast cancer cells.

## 2. Methods 

### 2.1. Materials

PTX was from Sigma (St. Louis, MO), sodium alginate from Cica-Reagent, Tokyo, Japan, and all other reagents were from Sigma-Aldrich or Merck. Water was purified using the Milli-Q reverse osmosis system (EMD Millipore, Billerica, MA).

### 2.2. Preparation of PTX-Loaded Alginate Nanoparticles by Nanoemulsification Polymer Cross-Linking

PTX was prepared by the nanoemulsification polymer cross-linking method. Briefly, 25 *µ*L of the drug (30 mg/5 mL), in 10 mL of chloroform, is emulsified under sonication in 30 mL of 0.1% M (W/V) aqueous solution of sodium alginate as emulsifier for 30 min and glycerol (15 mL) as stabilizer, and also emulsifier was added into the mixture. The mixture was cured for 24 h at room temperature (25°C). Nanoparticles were separated by ultracentrifugation (Cooling Centrifuge, Sigma, USA) at 10,000 rpm, 0°C for 10 min. To remove the nonincorporated drug, the obtained nanosuspension was filtered (S&S “filter paper circles,” pore size 1 mm). Nanoparticles were washed with Milli-Q reverse osmosis water and then dried at room temperature.

### 2.3. Preparation of Samples for TEM

TEM samples were prepared by drop coating the stock suspension on carbon-coated copper grids. Films placed on grids were air dried on tissue paper prior to measurement. TEM photograph shows nanoparticles (scale bar representing 500 nm, image taken at 80,000x magnification).

### 2.4. Cell Separation and Preparation

Breast cancer tumor tissue specimens were obtained at the time of surgery after informed written consent in accordance with South Egypt Cancer Institute ethical committee guidelines. Tissue was obtained from adult women that had been newly diagnosed with breast cancer. Tumor tissues were immediately disaggregated mechanically using a 16-gauge stainless steel mesh [23].

### 2.5. Monoclonal Antibodies and Viability Stain

For analysis of apoptosis by flow cytometry, propidium iodide (PI) and FITC conjugated annexin-V (IQ products, Netherlands) were used with the appropriate mouse isotype controls (BD PharMingen, San Diego, CA). All antibodies were used at concentrations titrated for optimal staining and carried out using standardized protocols.

### 2.6. Cell Cycle Analysis by Flow Cytometry

After treatment with anticancer drugs as indicated, breast cancer cells were fixed and permeabilized with 70% ice-cold ethanol for at least 1 h and then washed twice in phosphate-buffered saline (PBS). DNA was stained by incubating cells at 37°C for 1 h in 40 *µ*g/mL PI and 100 *µ*g/mL DNase-free RNase in PBS. As the fluorescence area (FL2-A) is the main parameter in the cell cycle analysis, histogram plots of FL2-A served as a cell cycle graph using Modfit LT 3.0 software (BD, San Jose, CA, USA) for analysis. Cells were analyzed for expression of annexin-V and both annexin-V and PI to identify early and late apoptosis, respectively.

### 2.7. Quantitation of Apoptosis by Flow Cytometry

Flow cytometry was performed with fluorescence-activated flow cytometry (FACS) Calibur system (BD, San Jose, CA). Fluorocytometric results were subsequently analyzed and displayed with CELL QUEST software (BD, San Jose, CA). Each analysis included measurements from a minimum of 10,000 cells.

### 2.8. Transmission Electron Microscopy

The morphological examination of nanoparticles was performed using TEM. The tungsten-filament 100 kV advanced high performance JEOL JEM-100CXII system (JEOL, Tokyo, Japan) was used to obtain high-resolution TEM images.

### 2.9. UV/VIS Spectroscopy

Absorption spectra of solutions were recorded using a Spectro UV-VIS Double Beam PC Scanning Spectrophotometer UVD-2950 (Labomed Inc., Los Angeles, CA) with 1 cm Stoppard quartz cells. The computer data system UVWin5 Software v5.0.5 was used to measure wavelengths and absorbance.

### 2.10. High Performance Liquid Chromatography-Fluorescent Detector (HPLC-FLD)

HPLC analysis was carried out using Agilent HPLC 1200 (Santa Clara, CA) system consisting of degasser, quaternary pump, and a fluorescence detector. HPLC chemstation software (Agilent) was used for instrument control, data acquisition, and data analysis. PTX, ALG, and ALG-PTX nanoparticles were eluted using a Zorbax Extend C18 column (150 × 4.6 mm ID × 5 *µ*m particle size) and detected at 232 nm excitation and 353 nm emission with a flow rate of 1.5 mL min^−1^. Column temperature was maintained at 25°C. The mobile phase consisted of 40% of water, 35% acetonitrile (ACN), and 25% methanol. pH was adjusted using a Jenway 3505 pH meter (Burlington, NJ).

### 2.11. Measure of the Nanoparticle Zeta Potential by Capillary Electrophoresis (CE)

Nanoparticles were prepared and the zeta potential (*ζ*) was measured using an Agilent 1600 CE system (Agilent Technologies, Germany), equipped with a diode-array UV/Vis detection system (190 to 600 nm). CE chemstation software was used for instrument control, data acquisition, and data analysis. Bare fused-silica capillaries (Agilent) are with 64.5 cm total length, 56.0 cm effective length, and 50.0 *µ*m internal diameter. The applied voltage was maintained at 20 kV with a controlled temperature at 25.0°C to give the current value of 15 *µ*A. A small amount of PBS was added to obtain charges in the solution and to mimic the ionic concentration in the human body. A total H_2_O : PBS ratio equal to 20 : 1 was used. This solution is then added to the cell to measure *ζ*. A new capillary was conditioned by rinsing with 1.0 mol L^−1^ NaOH for 40 min at 40.0°C and water for 20 min at 25.0°C. The flushing procedure was optimized to give precise analysis by flushing with 0.1 mol L^−1^ NaOH for 2 min, followed by water for 2 min, and then equilibrated with running buffer for 5 min. The procedure was repeated between runs in order to maintain sample purity.

## 3. Results

### 3.1. Characterization of PTX-Loaded ALG Nanoparticles

**Figure 2:**
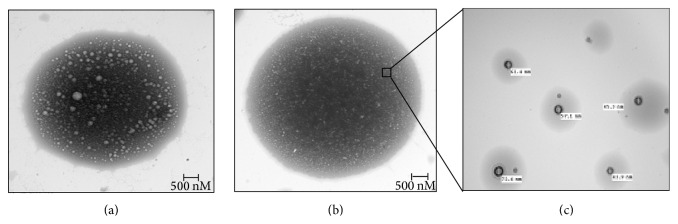
TEM images obtained for purified nanoparticle preparations. (a) HPLC isolated ALG nanoparticles. (b) HPLC isolated PTX-ALG nanoparticles. (c) High-power view of (b) inset represents high-power magnification view of the PTX-ALG nanoparticles.

**Figure 3:**
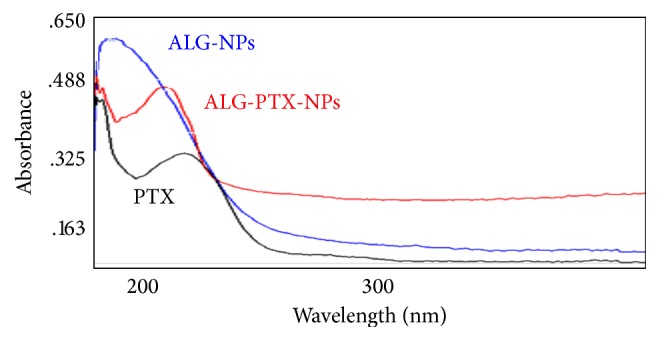
UV/VIS spectra of (a) ALG nanoparticles (blue curve), (b) PTX alone (black curve), and (c) ALG-PTX nanoparticles (red curve).

The size of nanoparticles in the stock dispersion was determined by TEM. Immediately after sonication and stabilization, Figure 2(a) shows the transmission electron microphotographs of the ALG nanoparticles and Figures 2(b) and 2(c) show the PTX-loaded ALG nanoparticles. Minimal drug degradation (0.1%) was seen after storage at 4°C for 8 months to indicate that the PTX-loaded ALG nanoparticles remained stable in polymer-generated matrices.  Figure 3 indicates the spectrophotometric analysis of PTX, ALG nanoparticles, and PTX-ALG nanoparticles. A shift is observed for PTX-loaded ALG nanoparticles (red) from that of PTX alone (black). Also it is shifted from the ALG nanoparticles alone (blue) to indicate the formation of a new compound that is detected between ALG nanoparticles and PTX.

### 3.2. HPLC Fluorescence

**Figure 4:**
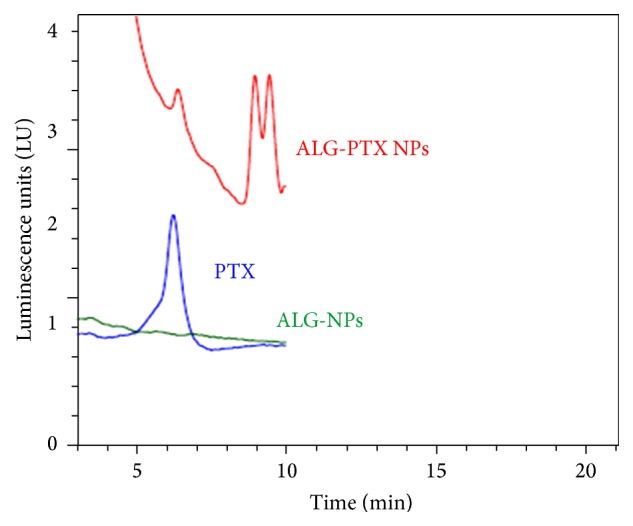
HPLC fluorescence for PTX alone (blue), ALG nanoparticles (green), and ALG-PTX nanoparticles (red). A Zorbax Extend C18 column (150 × 4.6 mm ID × 5 *µ*m particle size) was used for nanoparticles isolation with a mobile phase 40% of water, 35% acetonitrile (ACN), and 25% methanol and a flow rate of 1.5 mL min^−1^. Excitation was 232 nm and emission at 353 nm. LU represents luminescence units emitted.

Fluorescence spectroscopy is a type of electromagnetic spectroscopy, which then measures fluorescence within a given sample. Fluorescence spectroscopy provides valuable information regarding the interaction between PTX and the ALG. Size and shape of molecules can be determined using fluorescence spectroscopy. The interest of monitoring the fluorescence spectral changes is to study the interactions of PTX with the biodegradable molecules, for example, ALG. In these studies, we used liquid chromatography combined with fluorescence (Figure 4).

Zeta potential (*ζ*) is defined as the measure of magnitude for the electrostatic charge repulsion or attraction determined between two particles. Zeta is one known to affect stability and allows insight into the causes of dispersion and aggregation. To get information about the electrostatic potential of the particles in solution, as well as their colloidal stability, *ζ* of the prepared PTX-ALG nanoparticles have to be measured. A low *ζ* value is wanted in order for the particles to be as neutrally charged as possible and therefore less interference will occur* in vivo*. That will allow a better circulation all the way to the target site. At the same time a value near zero means instability and rapid coagulation. Colloidal stability is obtained at a value higher than +30 mV or lower than −30 mV. Also, a positive value is not desired because it is often a sign of toxicity. *ζ* is determined by measuring the electrophoretic mobility, which is the velocity of the particles when an electric field is applied to them. Indeed, if a particle is charged, it will move with a constant velocity (after equilibrium) to the electrode of opposite charge, when near electrolytes. The magnitude of the zeta potential, *ζ*, is given by

(1) $$\begin{array}{c} \zeta =\frac{4\pi \eta {µ}_{eo}}{\varepsilon }, \end{array}$$

where *η* is the viscosity, *µ*_eo_ the electroosmotic mobility, and *ε* the dielectric constant. *ζ* is calculated as –35 mV. The internalization efficiency and targeting ability of ALG nanoparticle were demonstrated by flow cytometry. PTX-loaded ALG nanoparticles showed a significantly higher cytotoxicity than commercially available PTX (Taxol).

**Figure 5:**
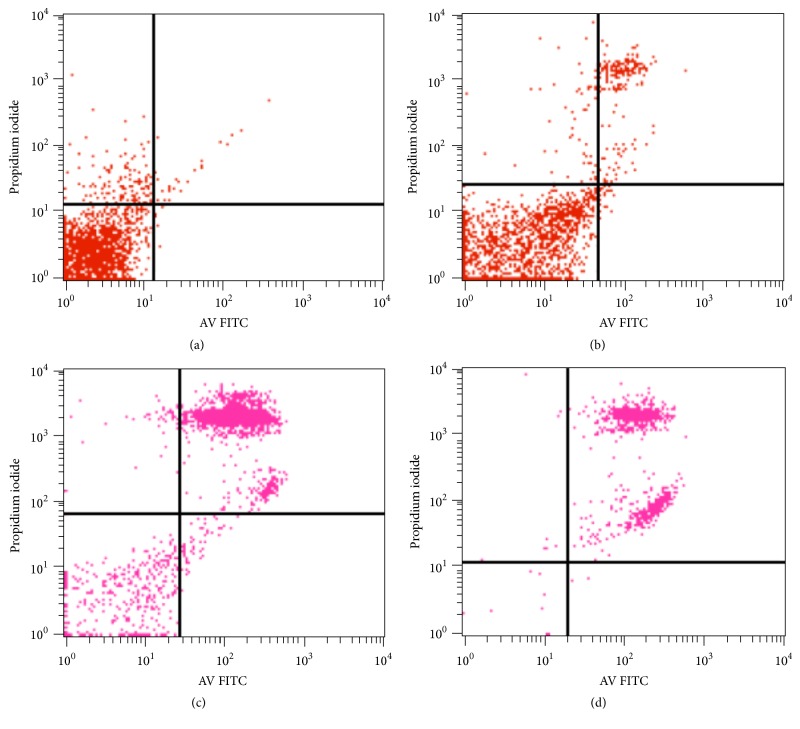
Dot plot of PI/annexin-V FITC-staining of breast cells. (a) Staining using the isotypic control antibody. (b) Staining of untreated breast cancer patient primary tumor cells. (c) Staining of primary breast cancer patient tumor cells after treatment with PTX. (d) Staining of breast cancer patient primary tumor cells with ALG-PTX nanoparticles.

Chemotherapeutics have been shown to inhibit cellular proliferation, block cell cycle progression, and induce apoptosis in cancer cells. Therefore, we evaluated the effect of PTX-ALG nanoparticles on the cell cycle and apoptosis of breast cancer cells using PI and annexin-V staining. Shown in Figure 5 are the results of one such experiment. The administration of PTX-encapsulated ALG nanoparticles led to tumor cell apoptosis and block in the cell cycle, compared with that observed with Taxol. We determined that the percentage of apoptotic cells in untreated cells was 11% and was increased to 83% after treatment of the breast cancer cells with PTX nanoparticles. Treatment with ALG-PTX nanoparticles further increased the percentage of apoptotic cells to 97%.

**Figure 6:**
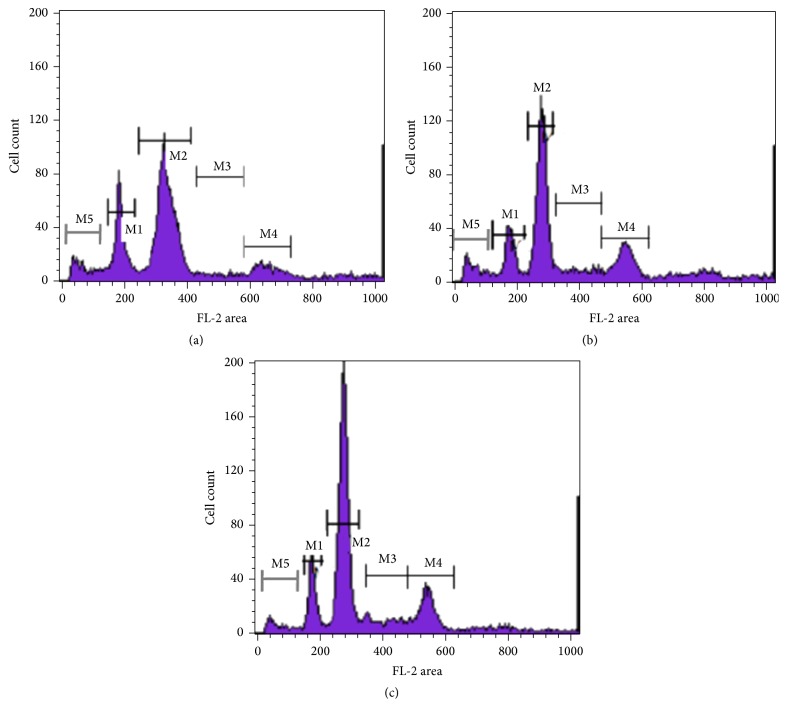
DNA content and cell cycle analysis of (a) control (untreated) breast cancer patient primary tumor cells, (b) breast cancer patient primary tumor cells treated with PTX for 24 h, and (c) breast cancer patient primary tumor cells treated with PTX-ALG nanoparticles for 24 h. M1 gate indicates the G0-G1 phase of diploid cells, M2 gate indicates the G0-G1 phase for the hyperdiploid cells, M3 gate indicates the S phase and M4 gate indicates the G2-M phase for the hyperdiploid cells, and M5 gate indicates the sub-G0-G1 (apoptotic) population. The S and G2-M phases of diploid cells were masked by the phases of hyperdiploid cells. All analysis of cell cycle was after gating of living cells to exclude most apoptotic cells.

Cell cycle analysis was performed to determine the effect of the nanoparticles on the cell cycle of patient primary breast cancer cells freshly isolated by tissue biopsy. Cell cycle arrest in the G2 phase was observed with PTX nanoparticles alone. A high amount of debris was detected making the exact percentage of cells in each phase difficult to be calculated by Modfit software. After treatment with PTX-ALG nanoparticles, cell cycle arrest in G2 phase was significantly increased (*P* < 0.05) (Figure 6).

## 4. Discussion

We have shown that PTX-ALG nanoparticles inhibit breast cancer cell viability and offer major advantages over PTX or PTX nanoparticles alone. PTX-ALG nanoparticles may reduce the unwanted side effects of PTX, increase drug solubility and dispersibility, offer biocompatibility, improve conjugation with other bioactive molecules, and increase the thermal and chemical stability of the drug. The nanoparticles prepared here also demonstrate improved optical properties and photostability. Since hydrophilicity of nanoparticles is very important to disperse nanoparticles in biological (aqueous) systems with biocompatibility a valuable alternative to conventional drug delivery systems, the ease of synthesis of the nanoparticles reported here also plays an important role in future bioapplications.

Recently the microparticle properties on PTX release and* in vitro* and* in vivo* pharmacodynamics have been evaluated. Biodegradable polymeric nanoparticles, based upon* in vivo* stability and safety in human patients, may provide an improved delivery method for anticancer agents [17, 18, 24]. The release of cancer drugs from poly(lactide-co-glycolide) PLGA-containing particles is currently under study. PTX delivery using PLGA-derived nanoparticles resulted in a reduction in PTX clearance from the peritoneal cavity as well as higher tumor exposure to PTX, compared to a Cremophor solution of PTX [25]. Future studies will address the precise molecular mechanisms by which ALG-PTX nanoparticles inhibit cancer cell growth and induce apoptosis. Future studies will also investigate whether the findings reported here are applicable to other cancers. An in-depth mechanistic understanding of the interactions between nanoparticles and tumors will lead to new strategies that advance clinical development of ALG nanoparticles as an anticancer therapy.
